# Supplementary material for: DMRforPairs: identifying Differentially Methylated Regions between unique samples using array based methylation profiles
Source: BMC Bioinformatics. 2014 May 15;15:141. doi: 10.1186/1471-2105-15-141 (PMC4046028; doi:10.1186/1471-2105-15-141)
Supplement: Additional file 2 — DMRforPairs output for the comparison of A431-MCF7 and NA17018-NA17105. Please start from the HTML files in each folder. Available via the BMC Bioinformatics website. [file 1471-2105-15-141-S2.zip › 1394847754114233_MOESM2_ESM/A431_MCF7/figures/8509.pdf]

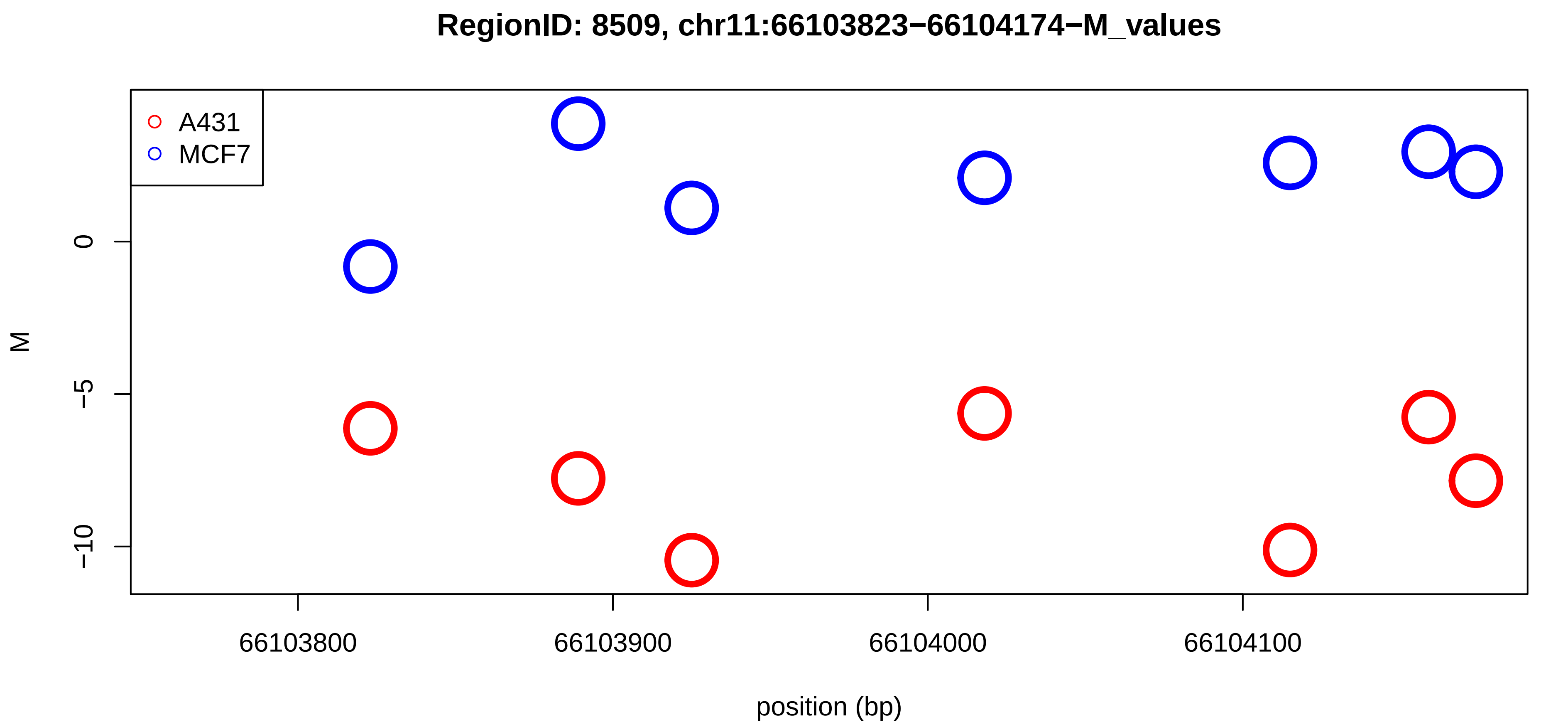
RegionID: 8509, chr11:66103823−66104174−M_values
A431
MCF7
M
0
−5
−10
66103800
66103900
66104000
66104100
position (bp)

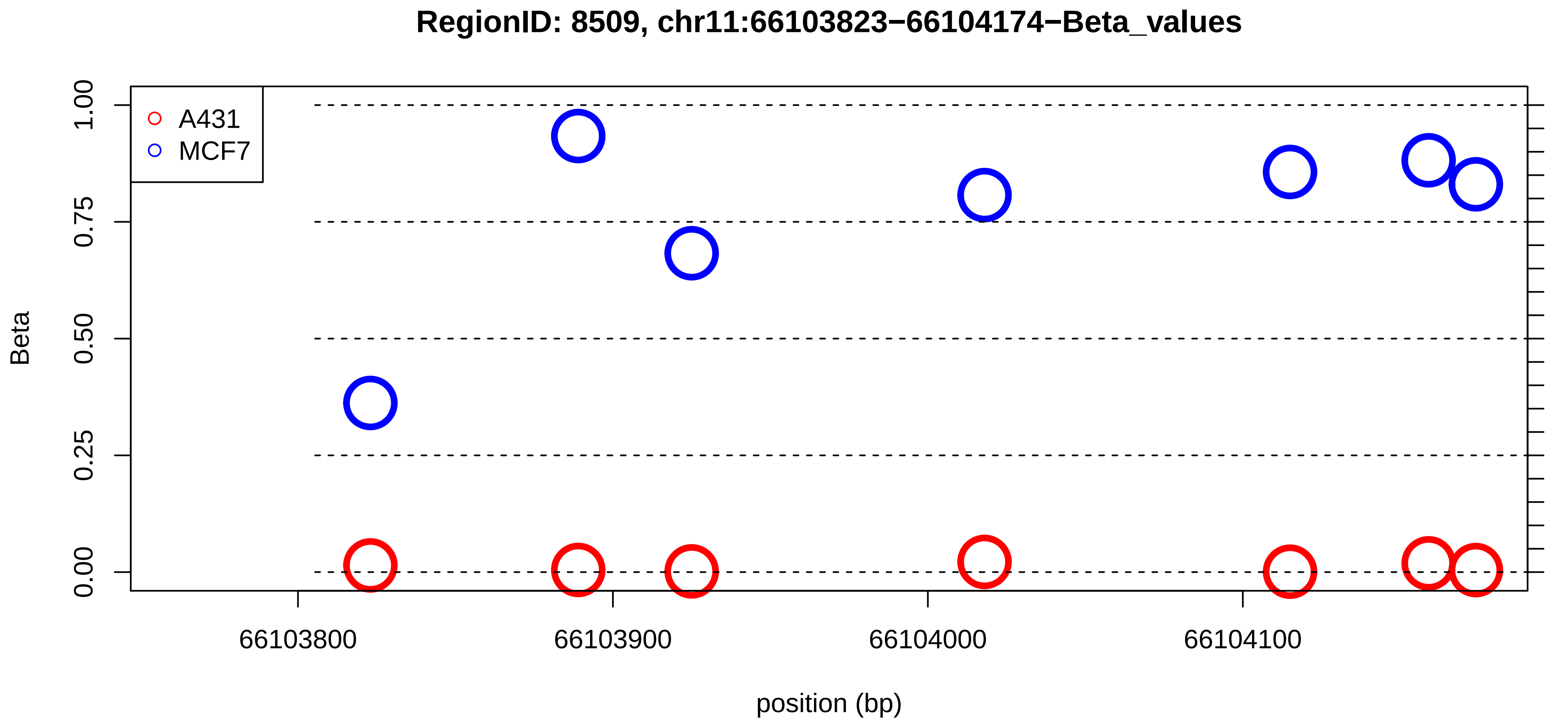
RegionID: 8509, chr11:66103823−66104174−Beta_values
A431
MCF7
Beta
1.00
0.75
0.50
0.25
0.00
66103800
66103900
66104000
66104100
position (bp)

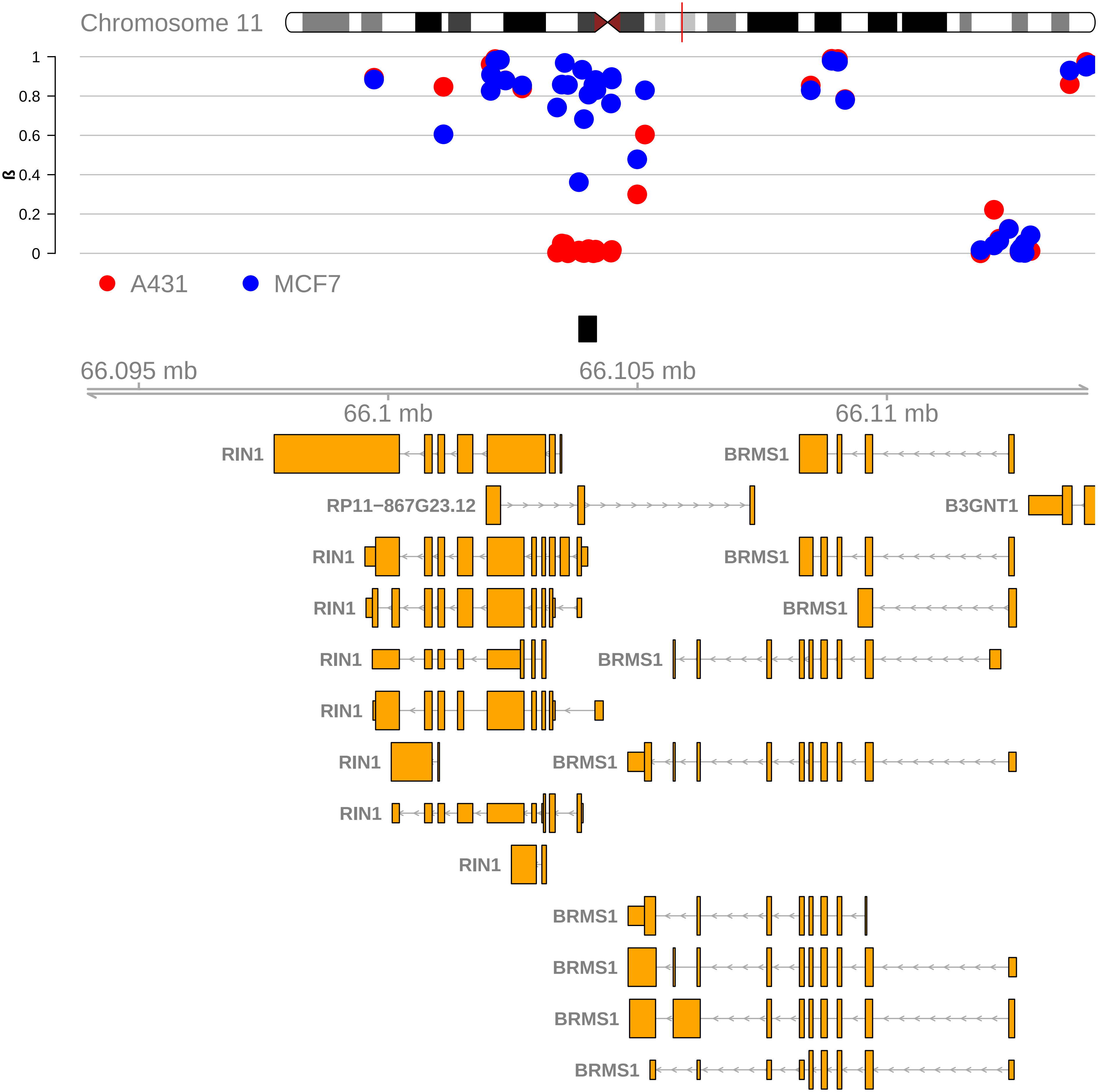

Chromosome 11
1
0.8
0.6
ß 0.4
0.2
0
A431
MCF7
66.095 mb
66.105 mb
66.1 mb
66.11 mb
RIN1
BRMS1
RP11−867G23.12
B3GNT1
RIN1
BRMS1
RIN1
BRMS1
RIN1
BRMS1
RIN1
RIN1
BRMS1
RIN1
RIN1
BRMS1
BRMS1
BRMS1
BRMS1
